# Evaluation of GPT-4’s Chest X-Ray Impression Generation: A Reader Study on Performance and Perception

**DOI:** 10.2196/50865

**Published:** 2023-12-22

**Authors:** Sebastian Ziegelmayer, Alexander W Marka, Nicolas Lenhart, Nadja Nehls, Stefan Reischl, Felix Harder, Andreas Sauter, Marcus Makowski, Markus Graf, Joshua Gawlitza

**Affiliations:** 1 Department of Diagnostic and Interventional Radiology School of Medicine & Klinikum rechts der Isar Technical University of Munich Munich Germany

**Keywords:** generative model, GPT, medical imaging, artificial intelligence, imaging, radiology, radiological, radiography, diagnostic, chest, x-ray, x-rays, generative, multimodal, impression, impressions, image, images, AI

## Abstract

Exploring the generative capabilities of the multimodal GPT-4, our study uncovered significant differences between radiological assessments and automatic evaluation metrics for chest x-ray impression generation and revealed radiological bias.

## Introduction

Generative models trained on large-scale data sets have demonstrated an unprecedented ability to generate humanlike text [1] and have performed surprisingly well on untrained tasks (zero-shot learning) [2]. In medical imaging, the applications are manifold, and it has been shown that models can not only draw radiological conclusions [3] but also structure reports [4] and even generate impressions based on the findings given in a report [5] or the image itself [6]. One of the leading obstacles limiting the development of models for generating clinically applicable reports is the lack of evaluation metrics that capture the core aspects of radiological impressions [7,8]. While there are initial studies on the perception of artificial intelligence (AI)–generated text in the general population [9], insights are missing for specialized areas such as medical imaging. Therefore, our study investigated the ability of GPT-4 to generate radiological impressions based on different inputs, focusing on the correlation between radiological assessment of impression quality and common automated evaluation metrics, as well as radiological perception of AI-generated text.

## Methods

### Overview

To generate and evaluate impressions of chest x-rays based on different input modalities (image, text, text and image), a blinded radiological report was written for 25 cases from a publicly available National Institutes of Health data set [10]. The GPT-4 model was given an image, the results, or both sequentially to generate an input-dependent impression. In a blind randomized reading, 4 radiologists rated the impressions based on “coherence,” “factual consistency,” “comprehensiveness,” and “medical harmfulness,” which were used to generate a radiological score based on a 5-point Likert scale of each dimension. Additionally, radiologists were asked to classify the origin of the impression (human, AI), providing justification for their decision. The text model evaluation metrics and their correlation with the radiological score were assessed. Lastly, common model metrics for text evaluation were extracted and compared to the radiological assessment. The supplementary methods in Multimedia Appendix 1 [5,8,10-17] provide further details.

### Ethical Considerations

Due to the publicly available data set used in this study, the requirement to obtain written informed consent from the participants was waived. Participants were anonymized.

## Results

**Figure 1:**
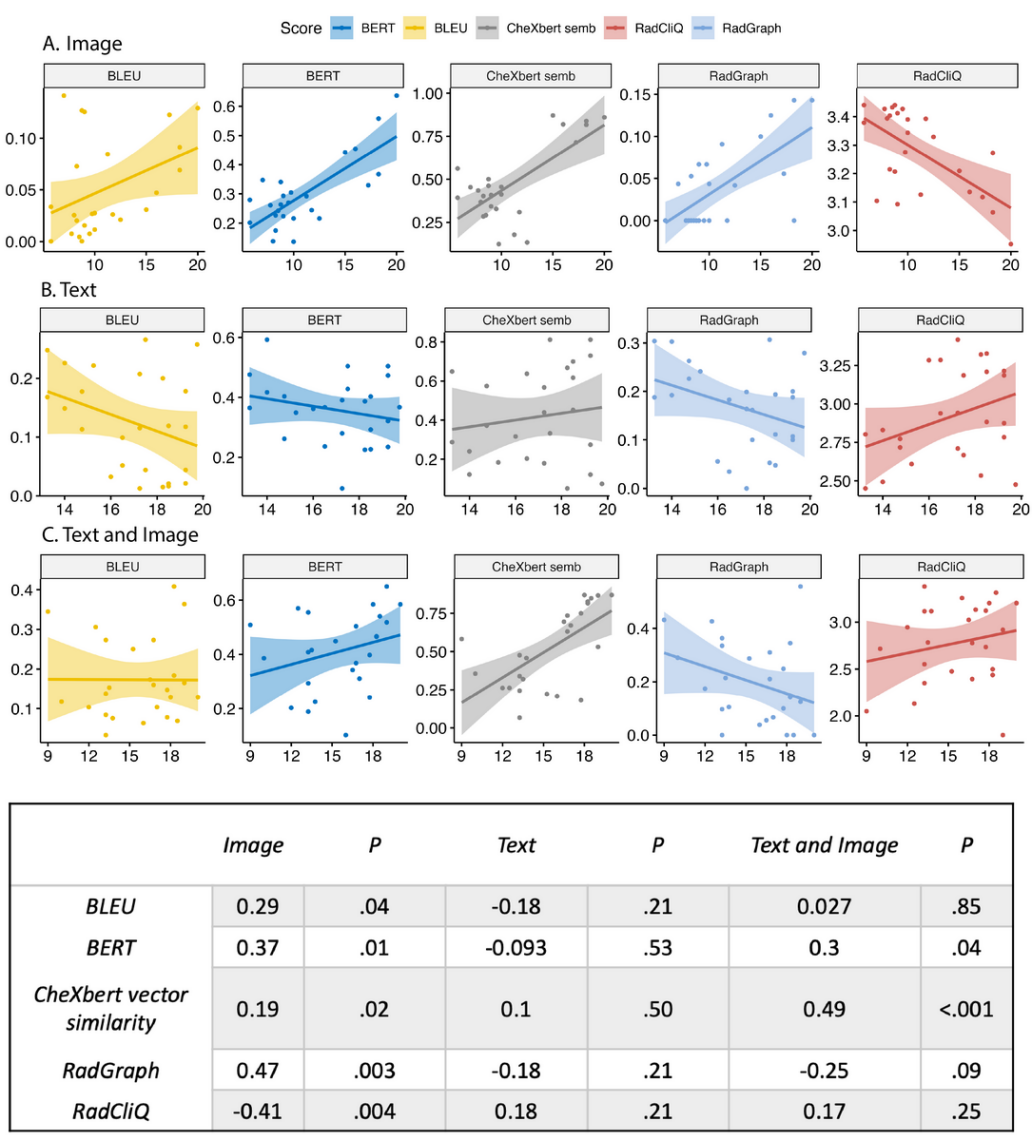
Scatterplots for each automated metric (BERT=blue; BLEU=yellow; CheXbert vector similarity=gray; RadGraph=light blue; RadCliQ=red) depending on the input: (A) image, (B) text, or (C) text and image. For the image input, all metrics except CheXbert vector similarity showed a significant correlation. However, the correlation was divergent or opposing for the text and text and image inputs. All correlation coefficients with their *P* values are shown in the lower section of the figure. BERT: Bidirectional Encoder Representations From Transformers; BLEU: bilingual evaluation understudy.

According to the radiological score, the human-written impression was rated highest, although not significantly higher than the text-based impressions (Table 1). A detailed analysis is shown in the supplementary results section in Multimedia Appendix 1. The automated evaluation metrics showed moderate correlations to the radiological score for the image impressions; however, individual scores diverged depending on the input (Figure 1). Correct detection of an impression’s origin (human/AI) varied by input (text: 61/100, 61%; image: 87/100, 87%; radiologist: 87/100, 87%; text and image: 63/100, 63%). For the text input, a homogeneous distribution was found, similar to radiological impressions classified as AI generated (supplementary figure in Multimedia Appendix 1). It was shown that impressions classified as human written were rated significantly higher by the radiologist, with a mean score of 18.11 (SD 1.87) for impressions classified as human written and 13.41 (SD 3.93; *P*≤.001) for impressions classified as AI generated.

**Table 1:** Quantitative and qualitative scores based on the input^a^.

	Qualitative			Quantitative		
	Radiologist score	BLEU^b^	BERT^c^	CheXbert vector similarity	RadGraph	RadCliQ
Image	10.97^d^	0.051^e^	0.298^e^	0.471	0.038^d^	0.328^d^
Text	16.95	0.125	0.356	0.417	0.168	0.291
Text and image	15.54^d^	0.173	0.411	0.523	0.197	0.278
Radiologist	18.47	N/A^f^	N/A	N/A	N/A	N/A

^a^Except for RadCiQ, which corresponds to the error rate, a higher score indicates a better approximation. For the automated metrics, the text and image–based impression score was highest, while the radiological score for the text-based impression was closest to the radiological ground truth.

^b^BLEU: bilingual evaluation understudy.

^c^BERT: Bidirectional Encoder Representations From Transformers.

^d^Indicates a *P* value <.05 for all higher input scores.

^e^Indicates a *P* value <.05 compared to the highest score.

^f^N/A: not applicable.

## Discussion

We evaluated the “out-of-the-box” performance of GPT-4 for chest x-ray impression generation based on different inputs. Based on the radiological score, text-based impressions were not significantly lower than the radiological impressions, whereas other inputs were rated significantly lower. Sun et al [5] showed that text-based impressions rated by radiologists were inferior. However, the study did not clarify if the radiological evaluations of the impressions were conducted under blinded conditions. Our work identified radiological bias, as impressions classified as human written received higher ratings. Therefore, without blinding, there is a risk that the inferiority of the AI-generated impressions is due to bias.

For the automated metrics, the impressions based on text and image were rated the closest to the radiological impressions, followed by text-based impressions. For the image-based impressions, there was a significant moderate correlation between the automated metrics and the radiological score; however, for the other inputs, opposite or nonsignificant correlations were found. Automatic metrics that capture relevant aspects of report quality are a prerequisite for successful development and clinical integration. Evaluation metrics, however, can only be as good as the human assessment, which is not free of bias and characterized by false heuristics [9]. Our findings underline this point, as impressions that were classified as human written scored significantly higher in the radiological assessment. Human evaluation is not error-free, but it is the benchmark for the evaluation of generated text.

Radiological heuristics, sources of error, and relevant aspects of radiological quality need to be further investigated, as they are essential for the development of useful model metrics.

## Appendix

Multimedia Appendix 1 Detailed methods, detailed results, and a mosaic plot visualizing the justification for classifying an impression as artificial intelligence generated.

## Abbreviations

AI: artificial intelligence

## Data Availability

The data sets generated and analyzed during this study are available from the corresponding author upon reasonable request.
