# Supplementary material for: Evaluation of GPT-4’s Chest X-Ray Impression Generation: A Reader Study on Performance and Perception
Source: J Med Internet Res. 2023 Dec 22;25:e50865. doi: 10.2196/50865 (PMC10770784; doi:10.2196/50865)
Supplement: Multimedia Appendix 1 [file jmir_v25i1e50865_app1.docx]

A

Methods:

Dataset and Report generation:

25 cases were randomly selected from the publicly available NIH Chest X-ray dataset, consisting of 100,000 de-identified images of chest radiographs with various pathologies [10]. Due to the publicly available dataset used in this study, the requirement to obtain written informed consent from the subjects was waived by the institutional review board. For each case a full report with “findings” and “impression” section was written by a blinded board-certified radiologist. Based on the outstanding “out-of-box” performance of GPT-4 and to allow for future comparison prompt engineering, for example retrieval augmented generation [11], chain-of-thought prompting [12] was reduced to a minimum. The only augmentation was done by including the word sequence “Let’s think step by step” in the prompt as it was shown to improve the zero shot capabilities of the mode [13]. For each case the chest x-ray image, the finding section from the written report and a combination of both was used as input. The following fixed prompt structure was used for all inputs {Imagine you are a radiologist. Generate a short radiological impression based on the main findings in (…). Use medical vocabulary. Let's think step by step.}. For each input the parentheses were specified as “text”, “image” and “text and image”.

Radiological reading:

A questionnaire adapted from Sun and Ong et al. was prepared and extended with the questions "Is the impression written by a human or an AI-model" and "What is the reason you think this is an AI-generated impression" [5]. The original questionnaire included four dimensions “Coherence”, “Factual consistency”, “Comprehensiveness” and “Medical harmfulness”, which were used to derive a radiological score based on a 5-point Likert scale for each dimension. Participants were given a short explanation for each dimension. For "Factual consistency" and "Medical harmfulness", a follow-up question was asked to specify the reasons why participants thought the impression was factually inconsistent and harmful. Each dimension was equally weighted resulting in a maximum score of 20 points and a lowest score of 4 points. The radiological score was recorded for each impression. Four radiologists from our institution (avg. experience in years = 7.7, range 3-12 years) rated the impressions based on the X-ray image and the finding section. The reading was done anonymously and blinded. Readers were presented with 4 randomly ordered impressions per case; the distribution of AI-generated and human-written impression was not known to the readers. For the question: "What is the reason you think this is an AI-generated impression" four choices were presented (“Error/Factual consistency”, “Spelling/Grammar”, “Structure/Coherence”, “Comprehensiveness”) readers were also able to give other reasons if the choices were not applicable.

Extraction of model metrics for text evaluation:

The extraction of the automatic metric to evaluate the generated impressions were based on the study of Yu, Endo and Krishan et al. [8]. The following metrics were extracted: BLEU [14], BERT [15], CheXbert vector similarity (CheXbert semb) [16], RadGraph [17] and the composite metric RadCliQ (Radiology Report Clinical Quality) [8], which combines BLEU and Radgraph. The BLEU score is calculated based on the number of matching n-grams (contiguous sequences of n words) in the predicted and reference texts. The score includes a penalty for predicted texts that are shorter than the references (brevity penalty). BERT score additionally tries to leverage contextual embeddings to include text semantics. Both the candidate (generated) text and the reference text are tokenized and fed into a pre-trained BERT model to obtain contextual embeddings for each token, which are then compared by calculating cosine-similarity and maximum similarity scores. CheXbert vector similarity and RadGraph are two scores that were developed to specifically evaluate radiological reports. CheXbert vector similarity defines a reference vector with the 13 most common labels (findings in a X-ray) and a no finding label and measures the cosine similarity. The Radgraph metric converts the texts into a knowledge graph with radiological dependencies and semantics and measures the overlap for both the generated and the ground truth text.. Lastly the proposed RadCliQ composition metric was extracted, which combines BLEU and Radgraph F1 (the harmonic mean of precision and recall) and is regarded as an estimate for the radiological error rate.

Statistical modelling:

All statistical modeling was done in Python and R. To evaluate the correlation between the automatic evaluation metrics and the radiological score, Kendall rank correlation coefficients were calculated. To compare each evaluation metric based on the input one-way ANOVA was calculated for all possible combinations. Significant ANOVA were followed up by Tukey post-hoc tests to perform multiple pairwise comparisons. Students t-test was used to compare the radiological scores between AI and human classified impressions. For all statistical tests a p-value below 0.05 was deemed significant.

B

Results:

Evaluation of generated and written Impressions:

For each score the impressions were analysed based on the input using ANOVA and Tukey post-hoc tests if the latter was significant. Except for CheXbert vector similarity, all scores showed significant differences in the ANOVA. For the automatic evaluation metrics the impression based on the combined text and image achieved the highest score, although not significantly higher than the text impressions (Turkey post-hoc tests text and image vs text: BLEU: difference=0.04 p=0.102, BERT: difference=0.05 *P*=0.31, CheXbert vector similarity: difference=0.11 *P*=.27, RadGraph: difference=0.03 p=0.61, RadCliQ: difference=0.14 *P*=.26). Image impressions were generally outperformed by text and text and image impressions, except for the CheXbert vector similarity (Turkey post-hoc tests image vs text/text and image: BLEU: difference=0.07/0.12 *P*≤.005/.0001, BERT: difference=0.06/0.11 *P*=.21/.006, CheXbert vector similarity: difference=-0.50/0.10 *P=*.71/.27, RadGraph: difference=0.13/016 *P*≤.0005/.0001, RadCliQ: difference=-0.36/-0.50 *P*≥.0005/.0001). Based on the radiological assessment the written impression by the radiologist achieved the highest score, although not significantly higher than text impressions (Turkey post-hoc tests radiologist vs text/text and image/image: difference= 1.52/2.93/7.50 *P*≤.23/.005/.0001).

C


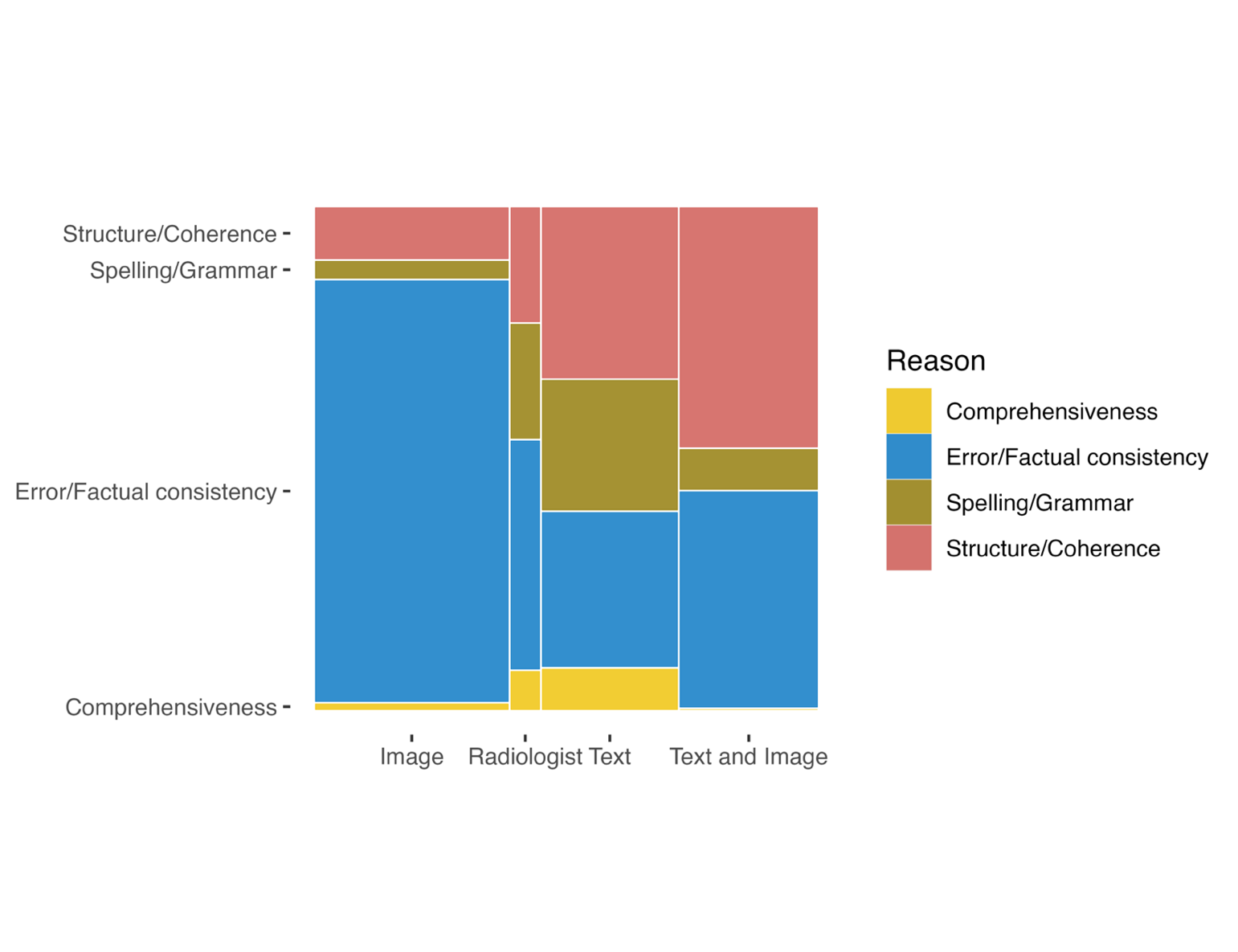


Mosaic-plot visualizing the justification for classifying an impression as AI-generated with the true origin on the x-Axis and the reasons on the y-Axis. The width of each bar corresponds to the frequency of being classified as AI-generated. The height of each colored bar corresponds to the percentage within the given origin.
